# Prevalence and impact of insomnia in patients with chronic obstructive pulmonary disease

**DOI:** 10.1177/14799731261490558

**Published:** 2026-09-18

**Authors:** Aureliano Ais-Daza, Angel Carmona-Gonzalez, Sofía Gutierrez-Hernandez, Isabel Castillo-Rivillas, Aurelio Arnedillo-Lopez, Aurelio Arnedillo-Muñoz

**Affiliations:** 1 Respiratory Department, 16824 Hospital Universitario Puerta del Mar, Cádiz, Spain; 2 Psychiatry Department, 16479 Hospital Clínico Universitario Lozano Blesa, Zaragoza, Spain; 3 INiBICA. Instituto de Investigación e Innovación Biomédica de Cádiz, Cádiz, Spain

**Keywords:** COPD, insomnia, quality of life, anxiety, depression, exacerbations

## Abstract

**Objectives:**

To determine the prevalence of insomnia in patients with chronic obstructive pulmonary disease (COPD) and evaluate its association with health status, pulmonary function, anxiety/depression, and exacerbations.

**Methods:**

A cross-sectional observational study was conducted including consecutive COPD outpatients. Data collected included sociodemographic and clinical variables, daytime sleepiness (Epworth Sleepiness Scale), quality of life (COPD Assessment Test, CAT), anxiety and depression (HADS), insomnia severity (Insomnia Severity Index, ISI), and exacerbations in the previous year. Multivariable logistic regression analysis was performed to identify independent predictors of insomnia.

**Results:**

A total of 103 patients (77 males, 26 females) were included. Insomnia prevalence was 38.8% (32.5% mild, 55.0% moderate, 12.5% severe). No significant differences in pulmonary function were observed between patients with and without insomnia. However, patients with insomnia had poorer health status (CAT 19.13±8.63 vs. 11.32±8.13; p<0.001), greater daytime sleepiness (Epworth 5.23±3.10 vs. 3.84±2.31; p<0.011), higher anxiety prevalence (61.5% vs. 31.2%; OR=3.533), and more exacerbations (50% vs. 28.6%; OR=2.5; p<0.037). In multivariable analysis, the CAT score was the only independent predictor of insomnia (OR 1.083; 95% CI 1.011–1.160; *p* = 0.023).

**Conclusions:**

Insomnia is highly prevalent in COPD and is associated with poorer health status, greater daytime sleepiness, increased anxiety, and more frequent exacerbations. Poorer health status was the only independent predictor of insomnia.

## Introduction

Chronic obstructive pulmonary disease (COPD) is a highly prevalent disease that has a substantial impact on patients’ quality of life. In Spain, the prevalence of COPD is 11.8% in the population over 40 years of age^1,^ and it is the fourth leading cause of death in this country, accounting for 6.95% of deaths.^2–4^

COPD extends beyond lung damage, has systemic implications and is associated with a high incidence of concomitant chronic conditions, which constitute comorbidities. Current estimates suggest that more than 80% of people diagnosed with COPD have at least one comorbidity, which can affect quality of life and increase the likelihood of exacerbations.^5–7^

Sleep disorders are important comorbidities in COPD patients. Up to 78% of patients present some type of sleep disturbance and previous studies have reported that the prevalence increases with the severity of airflow limitation,^8^ with the prevalence of insomnia being approximately 38.8%,^9^ although previous studies have reported prevalences of up to 50%.^10^ Insomnia in COPD patients is underestimated and underdiagnosed, and several factors may contribute to the development or worsening of insomnia, such as chronic cough, mucus production and side effects of COPD medication, such as corticosteroids and bronchodilators.^10^

Previous studies have reported that insomnia in COPD patients can have consequences for quality of life and even favor exacerbations and hospital admissions. Therefore, early diagnosis and treatment of insomnia are crucial to reduce the risk of adverse clinical outcomes.

The aim of this study was to analyze the prevalence of insomnia in a population of COPD patients and its potential impact on quality of life, anxiety and depression, and exacerbations.

## Materials and methods

### Design

An observational, cross-sectional study was conducted in consecutive patients with COPD attending the specialized COPD outpatient clinic at Hospital Universitario Puerta del Mar (Cádiz, Spain), a tertiary care hospital, between September 16, 2024, and November 20, 2024. COPD was diagnosed according to the current Global Initiative for Chronic Obstructive Lung Disease (GOLD) recommendations. The study followed the STROBE recommendations for reporting observational cross-sectional studies.

The exclusion criteria were the presence of other chronic respiratory diseases (e.g., asthma, interstitial lung disease, or bronchiectasis) or refusal to provide written informed consent. The diagnosis of COPD and the exclusion of other chronic respiratory diseases were confirmed by review of the patients’ medical records.

### Variables

Clinical and demographic data were obtained from the patients’ medical records. Patient-reported outcomes were assessed during the study visit under investigator supervision using validated questionnaires, including the Insomnia Severity Index (ISI)^11^ for insomnia severity, the Epworth Sleepiness Scale (ESS)^12^ for daytime sleepiness, the Hospital Anxiety and Depression Scale (HADS)^13^ for symptoms of anxiety and depression, and the COPD Assessment Test (CAT)^14^ for health status. Data were collected by two investigators according to a predefined standardized protocol to ensure consistency across all assessments. Pulmonary function tests were performed by experienced respiratory technicians in accordance with the current American Thoracic Society/European Respiratory Society (ATS/ERS) spirometry standards.^15^

The Hospital Anxiety and Depression Scale (HADS) contains two subscales with seven anxiety (HADS-A) and seven depression (HADS-D) symptoms, scoring from 0 to 3 each. A score of ≥ 8 is considered a clinically significant disorder, and a score of ≥ 11 suggests moderate–severe disorder.

The Insomnia Severity Index (ISI) is a validated index and is considered an instrument for assessing insomnia symptoms. It consists of seven items assessing falling asleep, difficulty maintaining sleep, waking too early, dissatisfaction with sleep quality, daytime impairments resulting from sleep disturbances, sleep problems noted by others, and distress caused by sleep difficulties. The total ISI score ranges from 0 to 28. Although ISI values greater than 7 points indicate the existence of insomnia, according to previous studies, the optimal cutoff point for the diagnosis of insomnia, with a sensitivity of 86.1% and a specificity of 87.7%, would be values greater than or equal to 10^16^; therefore, we considered this cutoff point for the diagnosis of insomnia in our study. The severity of insomnia was classified into four groups according to the final ISI score: no insomnia, ISI = 0–9; mild insomnia, ISI = 10–14; moderate insomnia, ISI = 15–21; and severe insomnia, ISI = 22–28.

The number of COPD exacerbations during the 12 months preceding study inclusion was collected from the patients’ medical records and the clinical interview conducted during the study visit. Exacerbations were defined according to the current GOLD recommendations.

### Sample size

The GRANMO online calculator (20) was used to calculate the sample size. The prevalence of insomnia in the general population (approximately 24%)^17,18^ was considered for the calculation of the sample size, and assuming an insomnia prevalence of at least 37% among patients with COPD, with an alpha error of 5% and a statistical power of 85%, we obtained a necessary sample size of 92 patients. Estimating a loss rate of 10%, we needed a sample size of 102 patients.

### Statistical methods

All analyses were performed using the Statistical Package for Social Sciences (SPSS v. 21.0; Armonk, NY, USA). Mean and standard deviation were used for normally distributed variables, while median and interquartile ranges were used for non-normal distributions. Categorical data are expressed as frequencies and percentages.

Clinical and demographic variables were compared between patients with and without insomnia using Student's t-test or the chi-square test. Variables showing a statistically significant association with insomnia in the univariate analysis were entered into a multivariable logistic regression model to identify independent predictors of insomnia. Given the sample size, the number of variables included in the model was deliberately restricted to reduce the risk of overfitting. P values <0.05 were considered statistically significant.

### Ethical and legal aspects

The present protocol was approved by the Health Research Ethics Committee of the Hospital Universitario Puerta del Mar (Cádiz). All participating patients provided written informed consent. The study was conducted following the international ethical recommendations contained in the Declaration of Helsinki (Fortaleza, 2013) of the World Medical Association, complemented, since 2016, by the Declaration of Taipei.

The collection, processing and communication of patients’ personal data was performed in compliance with the applicable legislation on personal data protection in Spain, as well as in Regulation (EU) 2016/679 of the European Parliament on Data Protection.

The data that support the findings of this study are not openly available due to reasons of sensitivity and are available from the corresponding author upon reasonable request. Data are located in controlled access data storage at Hospital Universitario Puerta del Mar (Cádiz).

## Results

A total of 106 patients were included, 3 of whom refused to participate in the study; thus, 103 patients were ultimately included in the analysis. The demographic characteristics are shown in Tables 1 and 2.

**Table 1.** Patient characteristics. Categorical variables.

​	N	Percentage
Sex		
Male	77	74,8%
Female	26	25,2%
Smoking status		
Exsmoker	74	71,8%
Active smoker	29	28,2%
GOLD stage		
A	38	36,9%
B	35	34,0%
E	30	29,1%
Dyspnea (mMRC scale)		
0	9	8,7%
1	33	32%
2	37	35,9%
3	20	19,4%
4	4	3,9%
Comorbidities		
Arterial hypertension	62	60,2%
Diabetes mellitus	38	36,9%
Dyslipemia	50	48,5%
Ischemic heart disease	22	21,4%
Arrhythmia	31	30,1%
Stroke	7	6,8%
Gastroesophageal reflux	2	1,9%
Asthma	4	3,9%
Obesity	47	45,6%
OSA	35	34,0%
OHS	8	7,8%
Insomnia	28	27,2%
Anxiety	26	25,2%
Depression	26	25,2%
Treatment		
Oxygen therapy	34	33,0%
CPAP	20	19,4%
NIMV	8	7,8%
Benzodiazepines	32	31,1%
Anxiolytics	25	24,3%
LABA o LAMA	10	9,7%
LABA+CI	9	8,7%
LABA+LAMA	31	30,1%
LABA+LAMA+CI	49	47,6%
ISI score		
ISI ≥ 10 Insomnia	40	38.8%
ISI 10-14 mild insomnia	13	32,5%
ISI 15-21 moderate insomnia	22	55%
ISI 22-28 severe insomnia	5	12,5%
Exacerbation in the previous year		
No	65	63,1%
Yes	38	36,9%

OHS: obesity hypoventilation syndrome. CPAP: continuous positive airway pressure. NIMV: non-invasive mechanical ventilation. LABA: Long-Acting Beta-Agonist. LAMA: Long-Acting Muscarinic Antagonist. CI: inhaled corticosteroid. ISI: Insomnia severity index.

**Table 2.** Patient characteristics. Numerical variables.

​	N	Media	Standard deviation
Age	103	70,80	7,69
Height (m)	103	1,65	0,08
Weight (kg)	103	78,67	17,03
BMI	103	28,97	5,94
Packs/year	103	54,57	23,01
FEV1 PBD (ml)	103	1383,20	583,88
FEV1 PBD (%)	103	52,76	18,37
FVC PBD (ml)	103	2344,27	821,44
FVC PBD (%)	103	68,98	19,43
Daytime sleepiness (Epworth)	103	4,38	2,72
Anxiety (HADS)	103	5,49	4,57
Depression (HADS)	103	4,58	4,27
Insomnio (ISI)	103	8,60	7,10
CAT	103	14,30	9,12

BMI: body mass index. FEV1: Forced expiratory volume in 1 second. FVC: Forced vital capacity. HADS: Hospital Anxiety and Depression Scale. ISI: insomnia severity index. CAT: COPD assessment test.

In the sample of COPD patients studied, the prevalence of insomnia was 38.8%. Insomnia was classified as mild or subclinical in 32.5% of the patients, moderate in 55% and severe in 12.5% (Table 1).

There were no differences in sociodemographic characteristics or spirometry values between patients with or without insomnia, except for working status, with active work status being related to having insomnia; however, given the small number of patients with active status, this result is probably not consistent (Table 4).

Patients with COPD and insomnia had significantly worse health status, as reflected by higher CAT scores, as well as greater anxiety and depression and greater daytime sleepiness (Tables 3 and 4).

**Table 3.** Comparison between COPD patients with and without insomnia. Numerical variables.

Variable	No insomnia (n=63)	Insomnia (n=40)	P value
Age	71.02±7.40	70.45±8.17	Ns
BMI	29.31±5.80	28.42±6.17	Ns
FEV1 (ml)	1343.49±600.08	1445.76±559.10	Ns
FEV1 (%)	50.84±18.05	55.77±18.69	Ns
FVC (ml)	2271.27±854.10	2459.25±763.46	Ns
FVC (%)	67.59±19.39	71.15±19.54	Ns
FEV1/FVC (%)	56.75±9.89	57.80±9.48	Ns
CAT	11.32±8.13	19.13±8.63	p<0.001
HADS Anxiety	4.19±3.96	7.53±4.77	p<0.001
HADS Depression	3.48±3.54	6.33±4.75	p<0.001
Epworth Scale	3.84±2.31	5.23±3.10	p<0.011

BMI: body mass index. FEV1: Forced expiratory volume in 1 second. FVC: Forced vital capacity. HADS: Hospital Anxiety and Depression Scale. ISI: insomnia severity index. CAT: COPD assessment test.

**Table 4.** Comparison between COPD patients with and without insomnia. Categorical variables.

Variable	No insomnia n=63	Insomnia n=40	Valor P
Seo			n.s.
- Male	47 (74.6%)	30 (75.0%)	​
- Female	16 (25.3%)	10 (25.0%)	​
Education level			n.s.
- None	5 (7.9%)	3 (7.5%)	​
- Obligatory	26 (41.3%)	15 (37.5%)	​
- High School	21 (33.3%)	9 (22.5%)	​
- University	11 (17.4%)	13 (32.5%)	​
Civil status:			n.s.
- Married	49 (77.7%)	35 (87.5%)	​
- Widower	7 (11.1%)	2 (5.0%)	​
- Single	5 (7.9%)	2 (5.0%)	​
- Divorced	2 (3.2%)	1 (2.5%)	​
Working status:			p<0.026
- Retired	61 (96.8%)	33 (82.5%)	​
- Active	2 (3.2%)	7 (17.5%)	​
Smoking status:			n.s.
- Active smoker	21 (33.3%)	8 (20.0%)	​
- Exsmoker	42 (66.7%)	32 (80.0%)	​
Dyspnea mMRC:			n.s.
- 0	6 (9.5%)	3 (7.5%)	​
- 1	19 (30.2%)	14 (35.0%)	​
- 2	24 (38.1%)	13 (32.5%)	​
- 3	12 (19.0%)	8 (20.0%)	​
- 4	2 (3.2%)	2 (5.0%)	​
GOLD:			n.s.
- A	​	12 (30.0%)	​
- B	​	14 (35.0%)	​
- E	​	14 (35.0%)	​
Comorbidities:			n.s.
- Arterial hypertension	36 (57.1%)	26 (65.0%)	​
- Diabetes mellitus	22 (34.9%)	16 (40.0%)	​
- Dyslipemia	29 (46.0%)	21 (52.5%)	​
- Ischemic heart disease	11 (17.5%)	11 (27.5%)	​
- Arrythmias	17 (27.0%)	14 (35.0%)	​
- Stroke	3 (4.8%)	4 (10.0%)	​
- Gastroesophageal reflux	1 (1.6%)	1 (2.5%)	​
- Obesity	29 (46.0%)	18 (45.0%)	​
- OSA	23 (36.5%)	12 (30.0%)	​
- OHS	6 (9.5%)	2 (5.0%)	​
- Asthma	0 (0.0%)	4 (10.0%)	p=0.021
Treatment			n.s.
Oxigen therapy	14 (22.2%)	6 (15.0%)	​
CPAP	6 (9.5%)	2 (5.0%)	​
NIMV	19 (30.2%)	13 (32.5%)	​
Benzodiazepines Ansiolytics	14 (22.2%)	11 (27.5%)	​
LABA or LAMA	4 (6.3%)	6 (15.0%)	​
LABA+CI	4 (6.3%)	5 (37.5%)	​
LABA+LAMA	23 (36.5%)	8 (20.0%)	​
LABA+LAMA+CI	29 (46.0%)	20 (50.0%)	​
Exacerbation in the previous year	18/63 (28.57%)	20/40 (50%)	p<0.037
Anxiety	10/63 (15.87%)	16/40 (40%)	p=0.01
Depression	9/63 (14.28%)	17/40 (42.5%)	p=0.002

OHS: obesity hypoventilation syndrome. CPAP: continuous positive airway pressure. NIMV: non-invasive mechanical ventilation. LABA: Long-Acting Beta-Agonist. LAMA: Long-Acting Muscarinic Antagonist. CI: inhaled corticosteroid. ISI: Insomnia severity index.

Patients with insomnia are 4.43 times more likely to have depression compared to those without insomnia (42.5% vs 14.28%; OR=4.43, CI90%: 1.72-11.39; p=0.002) and 3.53 times more likely to have anxiety (40% vs 15.87%; OR=3.53, CI90%: 1.40-8.91; p=0.01).

Similarly, according to the HADS questionnaire, the overall prevalence of anxiety was 25.2%, that of moderate–severe anxiety was 15.5%, and the prevalence of depression was 25.2%, with that of moderate–severe anxiety being 17.5%.

Patients with anxiety and depression had significantly higher CAT scores, indicating worse health status (CAT 20.31±9.25 vs 12.25±8.17 p<0.001 and (CAT 20.42±8.26 vs 12.21±8.47; p<0.001), respectively).

Patients with insomnia were more likely to have exacerbations in the previous year than patients without insomnia were (50% vs 28.57%, p=0.037; OR=2.50 CI95% 1.09-5.71) (Table 4).

No significant association was found between the occurrence of exacerbations during the previous year and benzodiazepine use, as the proportion of patients receiving benzodiazepines was similar among those with and without exacerbations (31.6% vs. 30.8%, respectively). Similarly, no significant association was observed for anxiolytic use (26.3% vs. 23.1%, respectively).

Variables that showed a statistically significant association with insomnia in the univariate analysis were entered into a multivariable logistic regression model (Table 5) to identify independent predictors of insomnia. Only the CAT score remained independently associated with insomnia. Each 1-point increase in the CAT score was associated with an 8.3% increase in the odds of insomnia (OR = 1.083; 95% CI, 1.011–1.160; p = 0.023).

**Table 5.** Multivariable logistic regression analysis for predictors of insomnia.

Variables	B	S.E	Wald statistic	Df	Sig.	OR
**CAT**	**.080**	**.035**	**5.183**	**1**	**.023**	**1.083**
HDA_A	.114	.067	2.860	1	.091	1.121
HDA_D	-.010	.080	.017	1	.896	.990
Epworth score	.121	.091	1.746	1	.186	1.128
N° exacerbations previous year	-.041	.274	.023	1	.880	.959
Constant	-2.807	.641	19.203	1	.000	.060

CAT: COPD assessment test. HDA_A: anxiety with the hospital anxiety and depression scale. HDA_D: depression with the hospital anxiety and depression scale. B: Regression Coefficient (Beta). S.E: standard error. df: degrees of freedom associated with the Wald statistic. Sig.: Statistical Significance (or p value). OR: odds ratio.

## Discussion

The results of this study showed that insomnia is highly prevalent among patients with COPD (38.8%) and is associated with poorer health status, greater anxiety and depression, increased daytime sleepiness, and a higher likelihood of exacerbations.

Sleep disorders in COPD patients are very common and vary according to the different studies and definitions of each disorder. Vaidya S. et al.^19^ reported that the prevalence of sleep disorders was 66% higher than in the control group, which was 16%, 31% of whom had two or more sleep disorders. In this study, insomnia was the most prevalent sleep disorder in COPD patients (49%) and was associated with anxiety or depression in 13% of the patients. The authors found no relationship between pulmonary function and insomnia, as was the case in our sample of patients; however, in another study, insomnia was related to worse pulmonary function.^20^

Other studies have reported that the prevalence of insomnia in COPD patients is between 23% and 53%.^21–27^ The results vary among the different studies analyzed, and this variability may be influenced by several factors. Nevertheless, its prevalence in patients with COPD is higher than in the general population. In Spain, there are no studies that have explored the prevalence of insomnia in patients with COPD, and the prevalence studies on sleep disorders in the general population are very limited and show that approximately 23% present some sleep difficulty and 11% report insomnia.^28^ These findings have already reported in young adults.^24^

Regarding the relationship of insomnia with anxiety and depression, previous studies^20^ have reported that the prevalence of anxiety and depression in COPD patients with insomnia is approximately 47%. In our study, the prevalence rates of anxiety and depression in the group of COPD patients with insomnia compared with patients without insomnia, were 40.00% vs 15.87% (p=0.001) and 42.50% vs 14.18% (p=0.002) respectively, i.e., like those reported in previous studies and were higher than those previously reported in unselected COPD populations.^29^

Consistent with Ban et al.,^30^ poorer health status assessed by the CAT was independently associated with insomnia (OR: 1.23; 95% CI, 1.13-1.34; p<0.0001). Although the estimated effect size differed slightly with our study (OR: 1.083; 95% CI, 1.011-1. 160; p=0.023), both support the CAT as a useful marker of insomnia risk in COPD.

Other studies have shown greater use of health care resources, such as more visits to the doctor, a higher rate of hospitalization, longer hospital stays, greater consumption of steroids and antibiotics, and a greater risk of exacerbations.^30–32^ In our study, we also observed that the probability of having experienced an exacerbation in the previous year was greater in patients with insomnia, but we did not explore the impact of insomnia on the use of healthcare resources, as in other studies.

Shorofsky M et al.^33^ followed a cohort of 480 COPD patients for 18 months and assessed sleep quality using the Pittsburgh Sleep Quality Index (PSQI). Patients with more exacerbations had significantly worse sleep quality. In our study, although another questionnaire was used, patients with an exacerbation in the previous year had higher scores on the ISI questionnaire than those who had not had exacerbations (7.08±6.45 vs. 11.21±7.47 p=0.004), indicating greater insomnia.

Our study has some limitations. First, its cross-sectional design precludes establishing causal relationships between insomnia and the evaluated outcomes. Second, this was a single-center study, which may limit the generalizability of the findings. Although the predominance of male participants reflects the epidemiological profile of COPD in Spain, it may also limit the applicability of the results to women with COPD. Third, while the sample size was adequate for estimating the prevalence of insomnia, it may have been underpowered for the multivariable logistic regression and some secondary analyses; therefore, these findings should be interpreted with caution and confirmed in larger multicenter studies. Finally, anxiety, depression, and insomnia were assessed using validated questionnaires rather than formal clinical evaluation, which may have resulted in some degree of misclassification.

In conclusion, in the analyzed population of COPD patients, there is a high prevalence of insomnia, which is associated with worse health status, as measured by the CAT questionnaire. Insomnia was also associated with greater daytime sleepiness, anxiety, depression, and a higher likelihood of exacerbations during the previous year.
